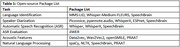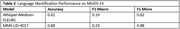# Global Research and Imaging Platform (GRIP): “FreeSurfer” for Digital Voice Processing

**DOI:** 10.1002/alz70856_102117

**Published:** 2025-12-25

**Authors:** Cody Karjadi, Huitong Ding, Mahdi Khemakhem, Ian Wong, Xavier Serrano, Edward Searls, Julia Peterson, Marisa Long, Katherine A. Gifford, Abhishek Pratap, Ting Fang Alvin Ang, Qiushan Tao, Philip Joung, Rhoda Au

**Affiliations:** ^1^ Boston University Chobanian & Avedisian School of Medicine, Boston, MA, USA; ^2^ Framingham Heart Study, Framingham, MA, USA; ^3^ Boehringer Ingelheim, Ingelheim am Rhein, Germany; ^4^ University of Washington, Seattle, WA, USA; ^5^ University of Toronto, Toronto, ON, Canada; ^6^ Institute of Psychiatry, King's College London, London, United Kingdom; ^7^ Slone Epidemiology Center, Boston University Chobanian & Avedisian School of Medicine, Boston, MA, USA; ^8^ Boston University School of Public Health, Boston, MA, USA; ^9^ Department of Pharmacology & Experimental Therapeutics, Boston University Chobanian & Avedisian School of Medicine, Boston, MA, USA; ^10^ Alzheimer's Disease Research Center, Boston University Chobanian & Avedisian School of Medicine, Boston, MA, USA, Boston, MA, USA

## Abstract

**Background:**

Digital voice is increasingly being recognized as a less biased and more scalable approach for identifying those with cognitive impairment in the early stages of Alzheimer's disease (AD) and other related dementias (ADRD). Just as brain MRI scans were widely adopted as a tool for in vivo detection of AD/ADRD, FreeSurfer was developed as an open‐source tool to facilitate brain MRI processing, opening the pathway for analysis and discovery. This toolkit seeks to similarly enable the usage of digital voice for AD/ADRD scientific advancement.

**Method:**

Global Research and Imaging Platform (GRIP) is developing an open‐access platform that will address common research pain points. In collaboration with GRIP, the first version of this toolkit has been published on a public GitHub repository. Table 1 lists the current voice processing tasks that can be accomplished via a variety of open‐source packages included in the toolkit, which have been tested on more than 33,000 Framingham Heart Study (FHS) recordings collected between 2005‐2024 and on several publicly available datasets. The toolkit contains detailed documentation of examples and containerized deployment via Docker for each package.

**Result:**

Language identification was performed on the MinDS‐14 dataset via existing models (Table 2). Speaker diarization on the VoxConverse dataset via the pyannote.audio package, resulting in the lowest diarization error rate (DER) of 9.0% among several tools. Automatic speech recognition (ASR) pipelines that utilize several available models and test on open‐source datasets (MinDS‐14, DisfluencySpeech) are included in the toolkit. ASR evaluation pipelines that produce metrics such as word error rate, match error rate, word information lost, word information preserved, and character error rate are also included. Acoustic features such as openSMILE low‐level descriptors and audio embeddings (Data2vec, Wav2Vec2) and natural language processing features including pauses and lexical diversity have been produced on FHS recordings (hour‐long neuropsychological tests, short smartphone‐app based).

**Conclusion:**

Digital voice may be an ideal scalable option for collection of cognitively relevant information in real‐world settings. The ongoing development of this modular toolkit will enable efficient and non‐proprietary processing of digital voice. GRIP will allow users worldwide, regardless of their technical expertise, to leverage robust digital voice processing pipelines.